# Characteristics of Japanese Elite Athletes Who Utilized Mental Health Services at the Japan High Performance Sports Center During the Tokyo 2020 Olympics and the COVID-19 Pandemic

**DOI:** 10.7759/cureus.86583

**Published:** 2025-06-23

**Authors:** Ritsuko Hashimoto, Tatsuya Yamaguchi, Kanami Suzuki, Kuniko Sekiguchi, Miho Tanaka, Kaori Eda, Anna Tomori, Kazuyuki Kamahara, Michiko Dohi, Koji Machidori, Kohei Nakajima

**Affiliations:** 1 Sports Medicine, Japan Institute of Sports Sciences, Tokyo, JPN; 2 Sports Medicine, Faculty of Medicine, Juntendo University, Tokyo, JPN

**Keywords:** covid-19, elite athletes, mental health, psychosomatic diseases, relative energy deficiency in sport

## Abstract

Background and objective

Reports on the mental health of Japanese athletes have been increasing in recent years, although many focus on specific sports, such as rugby. In this study, we hypothesized that the mental health of elite Japanese athletes was affected by the COVID-19 pandemic and the Tokyo 2020 Olympics, leading to a rise in mental health issues. The aim of this study was to clarify the impact of these events on the mental health of elite athletes and to identify trends and characteristics of those who sought care at the clinic.

Methods

We conducted a retrospective, cross-sectional observational study using medical records from the Sports Clinic at the Japan Institute of Sports Sciences (JISS), covering the period from January 1, 2019 to December 31, 2022. All athletes who received psychiatric or counseling services during this time were included. There were no exclusion criteria or age restrictions. Participant characteristics were analyzed using the chi-square test and Fisher’s exact test for categorical variables and the Welch t-test for continuous variables. All tests were two-tailed, with a significance threshold set at p < 0.05.

Results

A total of 85 athletes (23 men and 62 women) received treatment during the study period. Most of the patients visiting the mental health department at the JISS Sports Clinic were female athletes involved in individual sports, particularly in record-oriented disciplines. Since the onset of the COVID-19 pandemic, the number of athletes seeking mental health support has increased significantly. Reasons for consultation included both psychological issues, such as sleep disorders, and physical symptoms, such as gastrointestinal problems.

Conclusions

Mental health issues among elite Japanese athletes often manifest as psychosomatic symptoms. Recognizing this pattern is important for sports physicians, especially those without psychiatric training, and can support the development of effective care systems tailored to athletes’ needs.

## Introduction

It is widely accepted that participation in sports has a positive effect on mental health, contributing to a reduced risk of depression and generalized anxiety. However, the situation differs for elite athletes, who face the pressures of high-level competition and the constant demand for peak performance.

In recent years, mental health issues among athletes have attracted global attention. Athletes now have more opportunities to speak openly about their mental health struggles, reflecting growing public awareness and social interest in the topic. Surveys on the prevalence of mental health problems among athletes have shown rates that are comparable to or even higher than those in the general population [1-3]. According to Reardon et al., “prospective studies have reported that mental health disorders occur in 5% to 35% of elite athletes over a follow-up period of up to 12 months [4].

Mental health concerns in athletes represent a critical issue that requires attention. At our clinics, we provide mental health services for elite athletes, including psychological support and treatment by sports psychiatrists (hereafter referred to as “treatment”) as well as counseling by clinical psychologists (hereafter referred to as “counseling”) for various mental health disorders, problems, and psychological symptoms.

We speculated that while the Tokyo 2020 Olympic Games, scheduled to be held in Japan, would be a positive and motivational event for Japanese athletes, they would also pose a substantial psychological burden, regardless of whether athletes ultimately participated. We hypothesized that the number of athletes experiencing mental health problems would begin to increase around 2019, the year when athlete selection for the games began in earnest. Compounding this stress, the COVID-19 pandemic emerged during the same period.

The pandemic had a profound impact on athletes, both physically and mentally. With numerous sporting events canceled and athletes forced into lockdown, the disruption was significant. A survey on well-being conducted among Japanese university student-athletes in early 2021 found that the pandemic had affected both the physical and mental well-being of more than half of the respondents [5]. Additionally, training facilities were closed from April 8 to May 27, 2020, following the government’s declaration of a state of emergency. The Tokyo 2020 Olympic Games, originally scheduled for July 2020, were postponed to the following year. Given the mental health challenges observed worldwide during the pandemic, it is reasonable to assume that Japan’s elite athletes also faced considerable psychological pressure [6,7].

In recent years, an increasing number of reports have emerged on the mental health of Japanese athletes; however, many of these studies have focused on a single sport or include athletes from a broad range of competitive levels [5,8,9].

In this study, we investigated the trends and characteristics of athletes who visited the Japan High Performance Sport Center (HPSC) Sports Medical Clinic. Using clinical medical records, our goal was to clarify the impact of the COVID-19 pandemic and the Tokyo 2020 Olympics on the mental health of elite athletes. We hypothesized that these events would negatively affect mental health and lead to an increase in mental health-related issues among elite Japanese athletes.

## Materials and methods

Study site

The Japan HPSC adopts an integrated approach that combines Olympic and Paralympic sports to enhance Japan’s international competitiveness. HPSC operates two main facilities: the Japan Institute of Sports Sciences (JISS) and the National Training Center, both of which offer high-quality training environments and support programs in sports science, medicine, and information. At the JISS Sports Clinic (hereafter referred to as “the clinic”), specialists provide services including treatment for sports injuries, disabilities, illnesses, athletic rehabilitation, psychological counseling, and nutritional support.

As the clinic does not have full-time psychiatrists or counselors, athletes requiring urgent psychiatric care are referred to specialized external medical institutions without being seen by the clinic’s mental health department first.

Study design, setting, and sampling

This retrospective, cross-sectional observational study was based on medical records from the JISS Sports Clinic, spanning the period from January 1, 2019, to December 31, 2022.

We collected clinical data from athletes who sought psychiatric treatment and counseling at the clinic. No statistical sample size calculations were performed for this study. The facility is used exclusively by designated elite athletes and their support teams, referred by sports organizations for training. Therefore, the study population consisted of elite athletes competing in the Olympic and Paralympic Games or those designated by specific national sports organizations.

The study complied with the Ethical Guidelines for Medical and Health Research Involving Human Subjects issued by the Ministry of Education, Culture, Sports, Science and Technology and the Ministry of Health, Labour and Welfare (March 23, 2021), along with the associated Guidance (April 16, 2021). All athletes gave informed consent for the use of their medical records in this study, and parental consent was obtained for participants under the age of 15. The study protocol was approved by the JISS Ethics Committee (approval number 2023-022).

Participants

We identified all elite athletes who visited the clinic and received either psychiatric treatment or psychological counseling during the study period. All such athletes were included in the study, with no exclusion criteria or age limitations. Typically, patients first visit either the internal medicine or the orthopedic department. If the attending physician determines that mental health care is necessary, either in conjunction with treatment for physical conditions or after excluding physical causes, the athlete is then referred to a psychiatrist or clinical psychologist for further care.

Data collection and measurements

The study focused on first-time patients who received psychological counseling or psychiatric consultation at the clinic between January 1, 2019, and December 31, 2022. Relevant data were extracted from clinical records at the JISS Sports Clinic.

Survey variables included continuous variables such as age, the number of individuals seeking consultations, and the number of sessions involving combined psychiatric treatment and psychological counseling. Categorical variables included sex, type of sport, whether the sport was individual or team-based, chief complaint at the initial visit, and the presence of concurrent time-loss injuries, such as postoperative conditions, fractures (including stress fractures), and muscle strains. Sports were categorized according to classification criteria established by the facility (Table 1).

**Table 1:** Classification of sports

Records	Scoring	Combat sports	Racket sports	Ball games
Canoeing	Archery	Boxing	Badminton	Baseball
Cycling	Artistic swimming	Fencing	Soft tennis	Basketball
Modern pentathlon	Dance sport	Judo	Table tennis	Beach volleyball
Rowing	Diving	Karate	Tennis	Football
Sailing	Equestrian	Kendo	N/A	Handball
Short track	Figure skating	Taekwondo	N/A	Hockey
Ski alpine	Golf	Wrestling	N/A	Rugby
Speed skating	Gymnastics	N/A	N/A	Softball
Sport climbing	Rhythmic gymnastics	N/A	N/A	Volleyball
		N/A	N/A	
Swimming	Shooting	N/A	N/A	Water polo
Track and field	Skateboarding	N/A	N/A	N/A
Triathlon	Ski freestyle	N/A	N/A	N/A
Weightlifting	Surfing	N/A	N/A	N/A
N/A	Trampoline	N/A	N/A	N/A

Statistical analysis

Statistical analyses were conducted using Microsoft Excel 2021 and EZR version 1.68 (released on 2024/6/30; Saitama Medical Center, Jichi Medical University, Saitama, Japan). EZR is a graphical user interface for R (The R Foundation for Statistical Computing, Vienna, Austria) and is a modified version of R Commander designed to include statistical functions frequently used in biostatistics [10].

The null hypothesis assumed no differences between sex or type of sport among athletes who visited the mental health department and that the number and proportion of athletes seeking mental health treatment remained consistent across years.

Participant characteristics were analyzed using Fisher’s exact test for categorical variables and the Welch t-test for continuous variables. To assess the distribution of mental health patients by sport type, a goodness-of-fit test was conducted. Additionally, a chi-square test was used to evaluate changes in the number of individuals receiving mental health consultations over time.

All statistical tests were two-tailed, and a p-value < 0.05 was considered statistically significant. A post hoc power analysis for the chi-square test was performed using G*Power version 3.1.9.6 for Mac OS.

## Results

During the study period, a total of 26,100 patients visited the clinic, comprising 11,590 males and 14,510 females. After excluding non-athletes (such as staff and others), the number of athlete patients totaled 25,860. This represents the number of athletes who received care at the clinic (Table 2). The breakdown of these 25,860 athletes by sport type is as follows: 7,778 in record-based sports, 4,493 in scoring sports, 7,258 in combat sports, 2,504 in racket sports, and 3,827 in ball games.

**Table 2:** Annual number of consultations

Year	Mental health	Other departments	Total
2019	137	8158	8,295
2020	180	5,683	5,863
2021	278	5,883	6,161
2022	395	5,146	5,541
Total	990	24,870	25,860

A total of 85 elite athletes (23 men and 62 women) received treatment and counseling during the study period, accounting for 990 sessions in total: 166 for men and 824 for women. The study population included one male and four female athletes who had been receiving outpatient care since before 2018.

**Figure 1:**
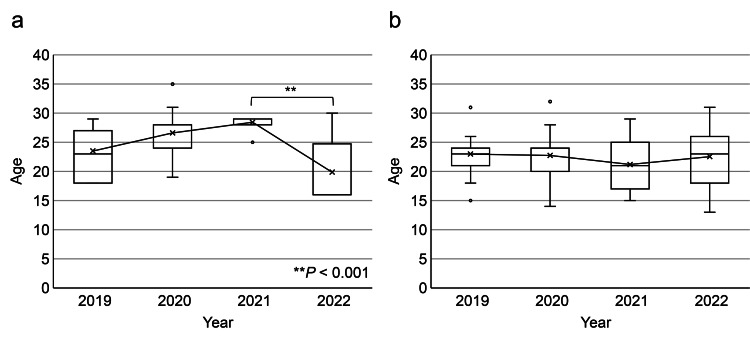
Age distribution of patients by year: (a) male and (b) female * P < 0.05; ** P < 0.01

The mean age of all athletes was 22.4 years (SD = 4.49). Male athletes had a mean age of 23.6 years (SD = 5.19; range: 16-35), while female athletes had a mean age of 22.2 years (SD = 4.31; range: 13-32). Female athletes were significantly younger than their male counterparts (t(213) = 3.17, P = 0.002). Notably, the average age of male patients decreased significantly from 2021 to 2022, dropping from 28.4 years to 19.9 years (t(75) = 12.24, P < 0.001) (Figure 1).

A significant difference was found in the number of patients presenting with mental health problems between 2019 and 2020, as indicated by a chi-square test (χ² = 30.932, df = 1, p < 0.001).

**Figure 2:**
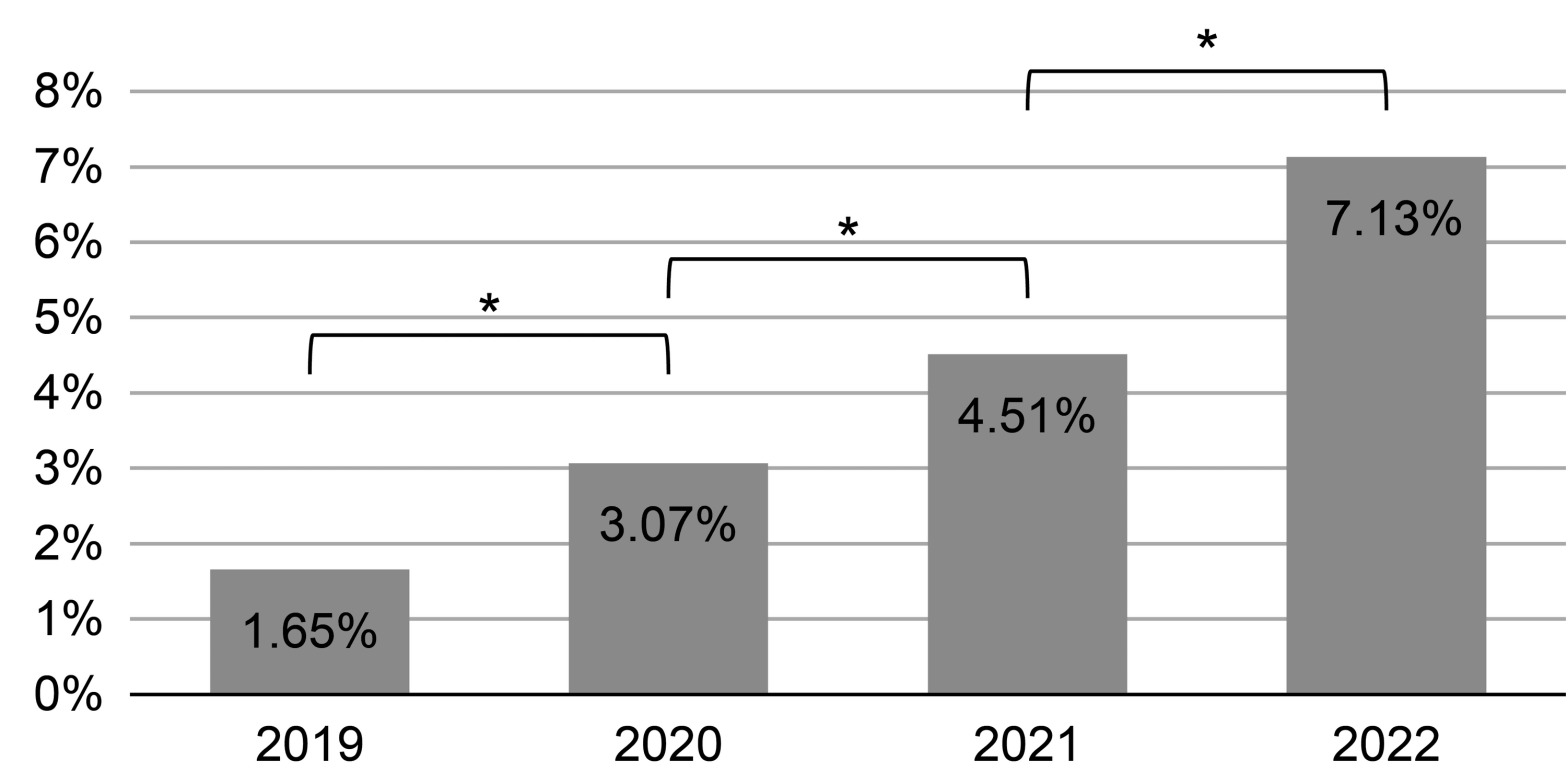
Percentage of mental health consultations relative to the total number of patients at the JISS Clinic * P < 0.05 JISS, Japan Institute of Sports Sciences

To examine the trend from 2019 to 2022, Fisher’s exact test was conducted to assess changes in the number of patients reporting mental health concerns. The test yielded a p-value of less than 0.01, indicating a statistically significant increase in the proportion of patients seeking mental health consultations over the years (Figure 2).

Of the total athletes, 75 (17 men and 58 women) were engaged in individual sports, while 10 (six men and four women) participated in team sports. Fisher’s exact test revealed a statistically significant predominance of female athletes in individual sports (P = 0.02) (Table 3).

**Table 3:** Number of patients by sports type

Category	Male		Female		Total
	Individual	Team	Individual	Team	
Records (endurance)	9 (5)	0	34 (13)	0	43 (18)
Scoring	3	0	10	1	14
Combat sports	4	0	11	0	15
Racket sports	1	0	3	0	4
Ball games	0	6	0	3	9
Total	17	6	58*	4	85

Further review of the clinical records showed that 18 of the 43 athletes were endurance athletes, including triathletes, middle- and long-distance runners, cyclists, open-water swimmers, and cross-country skiers (Table 4). 

**Table 4:** Details of records of athletes

Records		Male	Female
Canoeing		0	1
Cycling		1	2
Rowing		1	3
Sailing		0	1
Short track		0	2
Ski	Alpine	1	1
	Cross country	0	1
Sports climbing		0	2
Swimming	Open water	1	2
	Swimming	0	2
Track and field	Sprint	1	6
	Medium/long distance	3	6
	Jumping/throwing	1	4
Triathlon		0	1
Endurance		5	13
Total		9	34

To analyze the proportion of mental health patients by sport type at our clinic, we performed a goodness-of-fit test. The observed numbers of patients were as follows: records = 43, scoring = 14, combat sports = 15, racket sports = 4, and ball games = 9. The expected numbers, based on the overall distribution of athletes visiting the clinic, were as follows: records = 25.56, scoring = 14.76, combat sports = 23.85, racket sports = 8.23, and ball games = 12.57. The goodness-of-fit test yielded a p-value of 0.00 (α = 0.05), indicating a statistically significant difference between the observed and expected proportions (Table 3).

We also conducted a Fisher’s exact test to assess the association between sport type and sex (Table 3). The result was a p-value of 0.10, showing no statistically significant association at the α = 0.05 level. Post hoc power analysis for the chi-square test revealed a power of 0.62 (n = 85, effect size (Cramér’s V) = 0.31, α = 0.05, df = 4), suggesting that the lack of significant difference between males and females by sport type may be due to insufficient statistical power.

Table 5 presents the number of new patients and total sessions by year. Note that the facility was closed from April 8 to May 27, 2020, due to the government’s state of emergency declaration, resulting in no new patients being accepted during this period. In 2021, the year when the Tokyo 2020 Olympics were held, the number of new patients decreased because access was limited to Olympic athletes only. However, patient numbers increased again in 2022.

**Table 5:** Annual number of sessions

Year	Male		Female	
	New	Total	New	Total
2019	4	43	6	94
2020	6	41	23	139
2021	4	21	11	257
2022	10	61	30	334
Total	24	166	70	824

**Figure 3:**
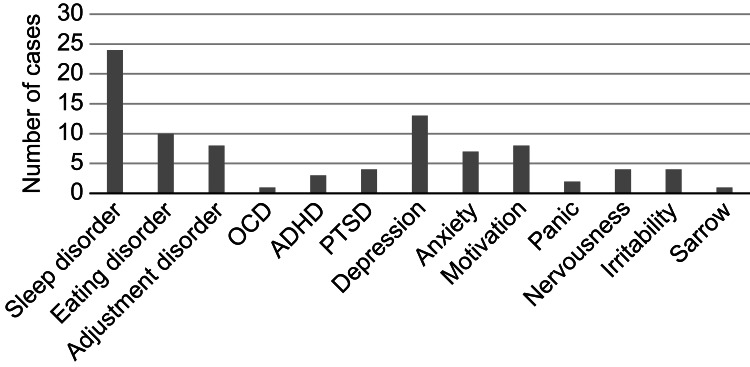
Mental health problems ADHD, attention-deficit/hyperactivity disorder; OCD, obsessive-compulsive disorder; PTSD, post-traumatic stress disorder

The complaints reported by patients during their first visit were categorized by sport type and tallied. When a patient presented multiple complaints, all were recorded and included in the tally. The most common issues were sleep disorders, followed by depression, gastrointestinal symptoms (such as stomach pain and nausea), concerns about family and interpersonal relationships, eating disorders, decreased motivation, worries about competitive performance, overtraining syndrome, fatigue, exhaustion, and anxiety. Among record-type athletes, the complaints were notably diverse. Endurance athletes in particular frequently reported physical problems, including overtraining syndrome and gastrointestinal symptoms (Figure 3, Table 6).

**Table 6:** Reasons for visiting our mental health department, categorized by sport type

Category		Subcategory	Record (endurance)		Scoring	Combat sports	Racket sports	Ball games	Total
Psychological	Diagnosis	Sleep disorder	14	-7	3	6	0	1	24
		Eating disorder	8	-5	1	0	1	0	10
		Adjustment disorder	3	-2	1	4	0	0	8
		Obsessive-compulsive disorder	0	0	1	0	0	0	1
		Attention-deficit/hyperactivity disorder	2	-1	0	1	0	0	3
		Post-traumatic stress disorder	2	0	0	1	0	1	4
	Symptoms	Depression	5	-2	3	3	0	2	13
		Anxiety	3	-1	2	2	0	0	7
		Motivation	5	-1	2	1	0	0	8
		Panic	0	0	2	0	0	0	2
		Nervousness	2	0	2	0	0	0	4
		Irritability	3	-2	1	0	0	0	4
		Sorrow	0	0	0	1	0	0	1
Physical	Diagnosis	Hyperventilation syndrome	2	0	0	0	0	3	5
		Overtraining syndrome	4	-3	0	1	0	0	5
	Symptoms	Gastrointestinal symptoms	9	-5	0	4	1	0	14
		Chest tightness and palpitations	0	0	2	2	1	1	6
		Fatigue	7	0	1	1	0	0	9
		Headache	2	0	0	2	0	0	4
		Appetite loss	2	-1	0	0	0	0	2
		Increased appetite	2	-1	1	0	0	0	3
		Dizziness and tinnitus	1	0	0	0	0	0	1
		Menstrual disorder	2	0	0	0	1	0	3
		Other	3	0	2	1	0	0	6
Social		Relationships	2	0	3	2	0	2	9
		Results and career	4	-1	1	3	1	0	9
		Practice environment	2	0	1	0	0	0	3
		Anxiety about the recurrence of injury or pain	0	0	0	1	0	1	2
		Harassment	0	0	1	0	0	0	1

Among the 85 patients, 15 (17.65%) had concurrent time-loss injuries. Specifically, four patients underwent surgery within the past year - two following anterior cruciate ligament reconstruction, one after medial patellofemoral ligament reconstruction, and one post-surgical treatment for a hip labral injury. Additionally, two patients experienced muscle strains, three had fractures or stress fractures (including lumbar spondylolysis), and one patient each was diagnosed with hamstring syndrome, shoulder impingement syndrome, shoulder tendonitis, patellar tendonitis, ankle sprain, and plantar fasciitis. Many patients also expressed concerns about injury recurrence, pain management, and anxiety related to rehabilitation.

## Discussion

In this study, we conducted a retrospective cross-sectional analysis of clinical data from Japanese elite athletes who sought mental health care both before and after the emergence of COVID-19. Our findings reveal the types and characteristics of mental health challenges faced by elite athletes in Japan. Importantly, this study focused on the factors that prompted athletes to recognize their mental health issues, rather than on psychiatric diagnoses, psychological evaluations, understanding of their conditions, or severity assessments.

Henriksen et al. have pointed out the need for a clearer definition of mental health in sports. While research often targets the prevalence of specific mental disorders such as depression, anxiety, and eating disorders, diagnostic labels may sometimes pathologize normal human experiences or overlook athletes who struggle with mental health problems but do not meet formal diagnostic criteria. Even without a clinical diagnosis, athletes may still require support to manage their mental well-being [11].

Our survey contributes new insights by highlighting the main concerns, diagnoses, and other issues experienced by Japanese elite athletes, framed within Henriksen’s conceptual perspective. Additionally, it identifies the sports and genders that appear more vulnerable to mental health problems.

A goodness-of-fit test comparing the observed and expected numbers of patients by sport showed a significant difference (P < 0.01), with record-oriented sports having a notably higher proportion of mental health patients than expected.

To summarize, more female than male patients visited the clinic during the study period. Also, a majority of the athletes were involved in individual sports, with a particular trend toward record-oriented sports.

Future research should investigate whether female athletes are generally more susceptible to mental health issues across larger populations and whether gender differences exist within specific sports. The predominance of individual sports and female athletes aligns with findings from previous studies [12-14].

Characteristics of female and individual sports elite athletes in Japan

Conditions such as depression, anxiety, and stress-related disorders are generally more prevalent among females in the general population [15-17]. This trend was also reflected among elite athletes in Japan, where most patients seeking mental health care were women.

Previous research on the impact of COVID-19 on athletes’ mental health found significantly higher rates of anxiety and depression among professional European soccer players during lockdown periods [6]. Moreover, athletes competing at higher levels tend to experience more severe mental health effects [12,13].

The higher prevalence of athletes from individual sports in our study may stem from fundamental differences in mindset and motivation compared to athletes in team sports. Pluhar et al. reported that individual sport athletes experience greater levels of anxiety and depression than those in team sports [14]. Melnyk et al. further demonstrated that individual and team sports present distinct mental health risks and protective factors: individual sports athletes tend to rely more on psychological well-being aspects like motivation and self-esteem, whereas team sports athletes benefit more from social well-being, including support from family and friends [18]. These contrasting factors may also influence how athletes adapt psychologically to environmental changes such as those caused by the COVID-19 pandemic.

Effect of the COVID-19 pandemic and Tokyo 2020 on Japanese elite athletes

Compared to the period before the COVID-19 pandemic and the Tokyo 2020 Olympics, consultations at the mental health department of our clinics increased significantly, a trend that continued even after the pandemic lockdowns. This rise may reflect how the pandemic and related disruptions affected athletes’ mental health and altered their perspectives. Previous studies have underscored the prevalence of mental health issues among elite athletes [1-4,6,9,11]. However, despite these challenges, elite athletes often show low willingness to seek mental health treatment due to multiple barriers such as stigma, limited mental health literacy, negative past experiences with treatment, and demanding schedules [19].

In 2019, the International Olympic Committee (IOC) issued a consensus statement on mental health [4], encouraging education and awareness programs targeting athletes and coaches. Proper mental health education and improved literacy have the potential to reduce stigma and change attitudes toward seeking treatment [20]. The COVID-19 pandemic brought health concerns beyond the infection itself, spotlighting the importance of mental health amid lockdown measures. This broader awareness may have been key in motivating athletes to pursue counseling or medical care.

Identifying stressors is often challenging; when these triggers remain unrecognized, various anxiety symptoms can emerge [21]. During the pandemic, many athletes struggled to pinpoint the exact causes of their anxiety and stress, with few directly attributing their mental health challenges to COVID-19 or the Tokyo 2020 Olympics.

Gouttebarge et al. identified COVID-19-specific stress factors such as health concerns and uncertainty about the future, which contribute to increased depression and anxiety among athletes [22]. The prevalence of generalized anxiety disorder (GAD) in elite athletes varies - 6.0% when clinically diagnosed and up to 14.6% based on self-assessment [23]. Similar to the general population, female athletes tend to report higher GAD symptoms than males [24]. While rates of GAD among elite athletes do not appear markedly different from the general population [25,26], adjustment disorders related to anxiety may be more common in this group [27].

Other studies show that anxiety linked to COVID-19 is significantly associated with sleep disturbances [28], that mental health declines during the pandemic occurred regardless of regional COVID-19 case numbers, and that younger individuals were especially vulnerable to stress, depression, and anxiety symptoms [29]. Among young athletes, depression and anxiety often manifest as physical symptoms such as headaches or gastrointestinal issues, highlighting the need for careful attention to these presentations [30].

In our study, during the 2020 lockdown, many athletes sought consultation primarily due to physical discomfort, more so than in previous or later years. Notably, most new patients were referred by the internal medicine department of the JISS clinic, meaning many initially presented with physical symptoms rather than overt psychological complaints. Gastrointestinal problems were common during this period, especially among endurance athletes, supporting previous suggestions that psychological stress and anxiety are linked to such symptoms in sports settings [31].

These findings imply that anxiety stemming from disrupted living conditions and uncertainties caused by the COVID-19 pandemic may have manifested as psychosomatic symptoms.

Additionally, periods of increased anxiety and depression risk in athletes include major injuries, career transitions such as retirement, and subsequent struggles with performance [32]. Our study found a significant decrease in the average age of male patients after the Tokyo 2020 Olympics, suggesting that this event served as a turning point for some athletes retiring or stepping back from competition.

Potential problems faced by athletes in record-oriented sports

In examining the details of athletes in record-oriented sports, we found that 18 of the 43 athletes were endurance athletes, including triathletes, middle- and long-distance runners, cyclists, open-water swimmers, and cross-country skiers (Table 4). Besides endurance sports, this group also included sports such as canoeing and sport climbing, where a lower body weight provides a competitive advantage. Consequently, many athletes in these record-oriented sports engage in high energy expenditure alongside strict weight control, often leading to a state of relative energy deficiency.

The female athlete triad (FAT) is a well-established condition characterized by menstrual irregularities and osteoporosis caused by insufficient energy availability. Previous research has reported a high prevalence of FAT in endurance and aesthetic sports [33-36]. For example, Nose-Ogura documented that amenorrhea is particularly common among Japanese female athletes in endurance and aesthetic disciplines, where strict weight management is frequently practiced [37].

Relative Energy Deficiency in Sport (RED-S) is a broader concept that includes FAT and recognizes the negative impact of energy deficiency on multiple physiological systems throughout the body. The IOC formally introduced RED-S in 2014 [38]. RED-S is associated with various pathological conditions, including decreased immunity, endocrine dysfunction, menstrual disturbances, osteoporosis, gastrointestinal issues, and mental health problems [39].

Our study found that many patients belonged to endurance or weight-sensitive sports, suggesting that RED-S might be a contributing factor to their health problems. Since RED-S can cause both physical and psychological symptoms, our observation that many athletes presented with psychosomatic complaints aligns with previous findings.

To effectively address mental health issues specific to athletes, collaboration between departments managing physical health and mental health specialists is essential.

Differences in mental health service utilization in Japan

Time-loss injuries can have a substantial impact on athletes’ mental health [40]. Research involving university football players shows that injured athletes are more likely to experience psychological issues, especially sleep disturbances [41]. Significant pain from injuries often leads to symptoms such as depression and anxiety [42]. Injured athletes tend to report more severe anxiety symptoms than their non-injured peers [2,24,43,44]. Consequently, psychotherapeutic approaches that address the psychological and mental health challenges arising from injuries or impairments are essential [40,42,45].

In general, people in Japan are less likely to seek professional help for mental health issues compared to Western populations. According to the 2023 Occupational Health and Safety Survey by the Ministry of Health, Labour and Welfare, 68.4% of respondents reported feeling more comfortable discussing work-related anxiety and stress with friends and family, whereas only 0.9% consulted a psychological counselor [46].

Among Japanese athletes, orthopedic surgeons often serve as the first point of contact for both physical and mental health concerns. However, although these surgeons may recognize mental health issues, they tend to discuss them less frequently with athletes compared to other professionals, such as physical therapists, and are less likely to refer athletes to mental health specialists [47].

Our approach, which facilitates access to mental health specialists through departments handling physical illnesses, might seem indirect. Yet, having non-psychiatric sports doctors, like orthopedic surgeons, who have frequent contact with athletes, act as bridges to mental health treatment may help reduce psychological barriers to seeking care. Shifting the mindset of these non-psychiatric sports doctors is necessary to achieve this goal.

We identified characteristics of elite Japanese athletes who sought mental health services in specialized sports medical institutions. However, further research focusing on different generations and competition levels is needed to determine whether these findings apply broadly to athletes across Japan.

Addressing athletes’ mental health requires not only expanding the number of sports psychiatrists but also changing the attitudes of existing sports doctors and the surrounding support network. There is an urgent need for sports organizations and healthcare providers to develop targeted mental health interventions, especially for female athletes and those in individual sports, who are at greater risk.

We recommend implementing regular mental health screenings and establishing support systems tailored to the unique challenges faced by these groups. Additionally, these systems should consider psychosomatic symptoms, such as gastrointestinal problems during periods of heightened stress like the COVID-19 pandemic, and promote integrated care models addressing both mental and physical health.

We hope our findings will be shared with sports doctors who are not mental health specialists, helping them to identify mental health problems in athletes early and facilitate timely referrals to appropriate specialists.

Limitations

This study was conducted with a select group of athletes, which limits the generalizability of the findings. A major limitation is the lack of diversity within the sample, as it did not account for variations in career stages or personal backgrounds, despite many athletes having lengthy careers.

Further limitations include the following. First, the eligible participants were restricted to elite athletes involved in the Olympics or specific domestic sports organizations. Consequently, elite athletes competing internationally or professionally in sports outside these categories were excluded from consultation opportunities. Second, there was a selection bias influenced by factors such as the competitive environment. Athletes training in regional cities may face logistical challenges that limit their ability to attend regular consultations, even if they hold elite status. Future research that encompasses a broader range of sports disciplines and geographical regions is necessary to address these limitations.

Lastly, since consultation reservations must be made through each sports organization, athletes wishing to keep their mental health concerns private may encounter barriers to accessing support. Understanding the characteristics of athletes who hesitate to seek help remains challenging. Thus, further studies are needed to explore the traits of elite athletes beyond those identified in this survey.

## Conclusions

This study characterized elite Japanese athletes who utilized mental health services at a specialized elite athletic healthcare facility. The majority of patients visiting the mental health department at the JISS clinic were female athletes involved in individual sports, with record-oriented sports being the most common category.

Mental health issues may underlie the physical complaints observed in female individual athletes and those engaged in record-oriented sports. Early intervention and prevention strategies focusing on the unique psychological traits of these groups could be effective. Nevertheless, further research is necessary to validate these findings within the broader population of elite athletes in Japan.
